# Cost-Effectiveness of Treatment Strategies for *BRAF*-Mutated Metastatic Melanoma

**DOI:** 10.1371/journal.pone.0107255

**Published:** 2014-09-08

**Authors:** Patti Curl, Igor Vujic, Laura J. van ‘t Veer, Susana Ortiz-Urda, James G. Kahn

**Affiliations:** 1 University of California San Francisco, San Francisco, California, United States of America; 2 The Rudolfstiftung Hospital, Vienna, Austria; 3 UCSF Helen Diller Family Comprehensive Cancer Center, San Francisco, California, United States of America; University of Tennessee, United States of America

## Abstract

**Purpose:**

Genetically-targeted therapies are both promising and costly advances in the field of oncology. Several treatments for metastatic melanoma with a mutation in the *BRAF* gene have been approved. They extend life but are more expensive than the previous standard of care (dacarbazine). Vemurafenib, the first drug in this class, costs $13,000 per month ($207,000 for a patient with median survival). Patients failing vemurafenib are often given ipilimumab, an immunomodulator, at $150,000 per course. Assessment of cost-effectiveness is a valuable tool to help navigate the transition toward targeted cancer therapy.

**Methods:**

We performed a cost-utility analysis to compare three strategies for patients with *BRAF+* metastatic melanoma using a deterministic expected-value decision tree model to calculate the present value of lifetime costs and quality-adjusted life years (QALYs) for each strategy. We performed sensitivity analyses on all variables.

**Results:**

In the base case, the incremental cost-effectiveness ratio (ICER) for vemurafenib compared with dacarbazine was $353,993 per QALY gained (0.42 QALYs added, $156,831 added). The ICER for vemurafenib followed by ipilimumab compared with vemurafenib alone was $158,139. In sensitivity analysis, treatment cost had the largest influence on results: the ICER for vemurafenib versus dacarbazine dropped to $100,000 per QALY gained with a treatment cost of $3600 per month.

**Conclusion:**

The cost per QALY gained for treatment of *BRAF+* metastatic melanoma with vemurafenib alone or in combination exceeds widely-cited thresholds for cost-effectiveness. These strategies may become cost-effective with lower drug prices or confirmation of a durable response without continued treatment.

## Introduction

Following advances in understanding of the genetics of tumor growth, several genetic testing-guided metastatic melanoma treatments have entered the market in recent years. Vemurafenib, the first drug specifically targeting *BRAF-*mutated melanoma, has become increasingly popular, and others have been developed in the wake of its success [1]. These therapies improve outcomes, but are expensive. Until 2010, the standard of care was chemotherapy with dacarbazine, with a median overall survival of 5.6 to 7.8 months after treatment initiation [2]–[4]. New therapies introduced since that time include ipilimumab (an immunomodulator), vemurafenib (a mutant-selective *BRAF* inhibitor approved for patients with a *BRAF^V600^* mutation), and most recently dabrafenib (a different *BRAF* inhibitor) and trametinib (a *MEK* inhibitor which is also approved for *BRAF*-mutated cancers). Ipilimumab was approved in 2010 and costs $150,000 for a full course [5]. It was shown to improve survival as a second-line therapy–when compared to a placebo-equivalent peptide vaccine–with a median survival of 10 months [6]. The combination of ipilimumab and dacarbazine also demonstrated improvement in survival over dacarbazine alone [7]. Vemurafenib was approved for the treatment of *BRAF*-mutated melanoma in 2012 after a phase III trial that reported a 0.37 hazard ratio for death in patients with *BRAF* mutations taking vemurafenib compared with dacarbazine, and a phase II trial showing 15.9 month median survival [8], [9]. The current cost of vemurafenib is $13,000 per month (for a total of $207,000 for a patient with median survival), and the cobas 4800 BRAF V600 Mutation costs $150 [5], [9]. Both dabrafenib ($9100 per month) and trametinib ($10,400 per month) were approved in 2013 based on improvement in progression-free survival compared to chemotherapy [5], [10], [11].

In clinical practice in the US, most metastatic melanomas are tested for *BRAF^v600^* mutations, and treated with a *BRAF* inhibitor if positive. For patients who progress while on a *BRAF* inhibitor, it is common to offer ipilimumab as a second-line treatment–although the benefits of this exact sequence have not been well studied. Although approved as monotherapy, trametinib on its own is not commonly used in clinical practice. Based on some encouraging phase II trial data, the combination of *BRAF* and *MEK* inhibitors is becoming more frequent in clinical practice, despite the lack of FDA approval of the combination and a limited amount of clinical data [12]. Although these new therapies increase duration of life, they have had limited effect on the overall prognosis in metastatic melanoma–likely due to limitations in their mechanisms of action. Specifically, targeted inhibitors of a single mutation are vulnerable to development of tumor resistance, and the benefit of immunomodulators is attenuated by melanoma’s ability to generate an immunosuppressive environment [13].

With the advent of beneficial and expensive new therapies, we believe it is essential to understand the differences among regimens in costs and clinical outcomes. We performed a cost-effectiveness analysis of genetic testing and targeted therapy for metastatic melanoma. We compare dacarbazine, vemurafenib alone, and vemurafenib followed by ipilimumab. In order to estimate the total costs of treatment (including follow-up) and expected health outcomes, we incorporated the best available data for each regimen into a decision model, considering both quality and duration of life. We thus arrive at estimates of the incremental cost and cost-effectiveness of the new regimens.

## Methods

### Study design

This cost-effectiveness analysis was designed according to the reference case in the guidelines of the Panel on Cost-Effectiveness Analysis in Health and Medicine [14]. The baseline analysis considered a population of treatment-naïve patients with *BRAF*-mutated metastatic or unresectable melanoma. The time horizon was the remaining life expectancy of the patients. All direct cancer-related medical costs for testing, treatment, monitoring, follow-up, and side-effect management were considered at 2013 prices [5], [15], [16]. The analysis was done from a societal perspective with a discount rate of 3%. Our primary outcome is the incremental cost-effectiveness ratio (ICER) between vemurafenib and dacarbazine. This value is acquired by dividing the total difference in costs between the two treatment strategies by the total difference in quality-adjusted life years (QALYs) between the two strategies. Our secondary outcome is the ICER between vemurafenib alone and vemurafenib followed by ipilimumab.

### Analytic model

**Figure 1:**
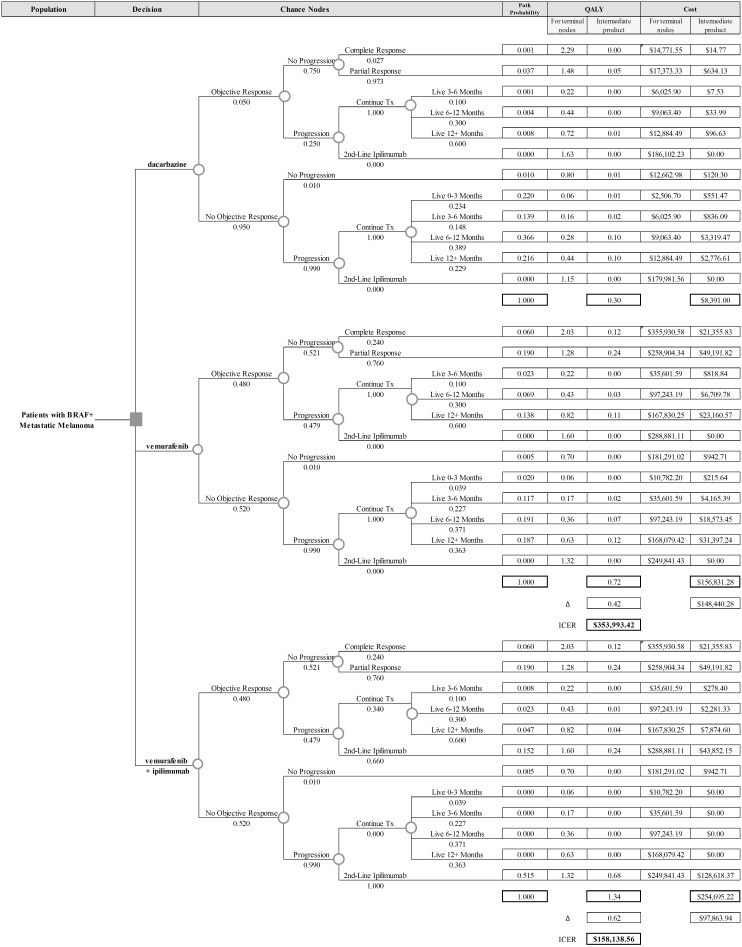
Decision Tree Model: The decision tree model used to estimate outcomes for the first year following treatment initiation. The decision nodes list the strategies modeled and the chance nodes list the probabilities of different clinical events during the first year. The path probability is the chance a patient will follow each specific path. The QALY and cost numbers in the “for terminal nodes” column is the average values over the course of a year for each patient taking that path, and the “intermediate product” column shows those individual averages adjusted for the proportion of people taking that path. Beneath these columns is the summation of the columns, representing to average costs and QALYs associated with the strategy. Beneath these averages in the second and third strategies are the differences and ICERs comparing each strategy to the strategy directly above it.

We created a deterministic expected-value cost-utility model to calculate the present value of all expected lifetime costs and all expected lifetime QALYs resulting from three potential treatment strategies–dacarbazine alone, vemurafenib alone, and vemurafenib with ipilimumab as a second-line therapy. We developed a decision tree model (Figure 1) to quantify outcomes in the first year following treatment initiation using data from pivotal clinical trials [6], [9].

**Figure 2:**
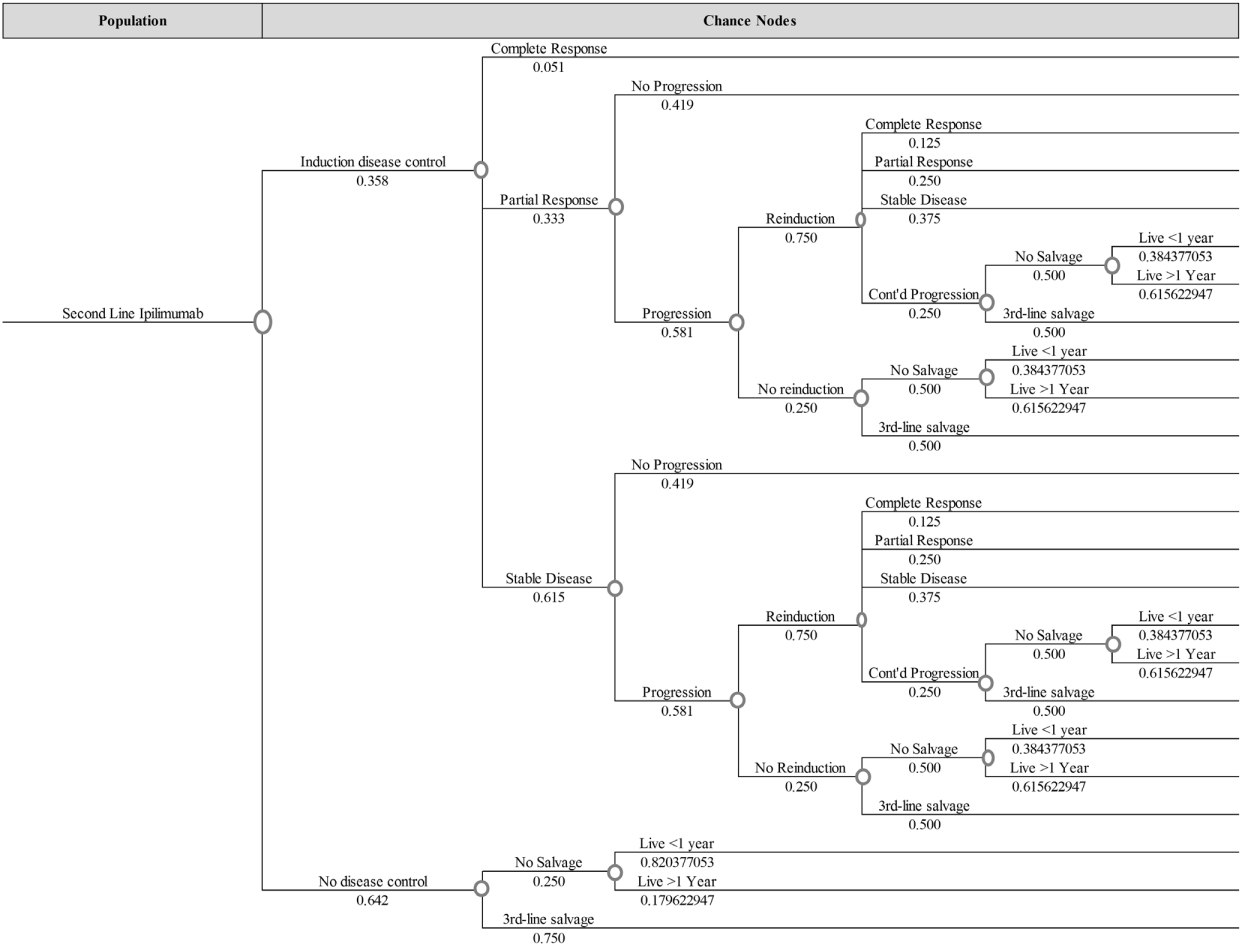
Decision Tree Model for Second-Line Ipilimumab: See format and conventions described in **Figure 1**.

**Figure 1** shows all branches of the decision tree model. The three treatment strategies modeled fill the decision column on the far left, and the tree branches out to the right with multiple chance nodes, dividing possible paths that a particular case might follow. Based on the definitions used in clinical trials, we first divided cases between those who had an initial objective response and those who did not. Next, cases were divided by whether they would experience progression within the first year of follow-up. For those with an objective response and no progression, we divided cases into those with a complete response and those with a partial response at the end of a one-year period. When quantifying outcomes for these cases, we assumed patients started in a stable disease state and remained in that state for the average time to response reported in clinical trials before transitioning to their final classification for the remainder of the first year. For cases which had an initial objective response but eventually progressed, we further divided by their duration of life following treatment initiation. When quantifying outcomes for these cases which died in the first year, our model assumes that they remained in stable disease for the average time to response then spent half of their remaining time alive in a partial response state and the other half in a progressive disease state. For the strategy of vemurafenib followed by ipilimumab, our model assumes that 100% of patients who progressed with no response to the initial therapy would switch, and 66% of patients who had an initial response but eventually progressed would switch at the time of progression. For patients who switched treatments, we assumed they switched at the first time progression was identified. We calculated outcomes after switching to ipilimumab in a second similar tree of paths following ipilimumab administration (Figure 2). The tree divides patients with no objective response between those who progressed within one year, and those who did not. We quantified outcomes for patients with no response or progression assuming they were in a stable disease state for the first year of treatment. We divided the patients who progressed without initial response similarly to those who progressed after response, adding an additional category of cases that died within the first three months of treatment. To quantify outcomes for these patients, we assumed they stayed in a stable disease state for the average time to progression reported in trials, and then spent the rest of their lives in a progressive disease state. For patients who lived less than the average time to progression, we assumed they spent half of their lives in the stable disease state and half in a progressive disease state. For clinical outcomes which were not explicitly divided between disease-state groups in the outcomes reported in the trials, we used coefficients that allowed differences between disease-state groups and averaged to the values reported in all patients. For example, mortality over a 3 month period was assumed to result from considerably more deaths in patients with progressive disease than those with stable disease or response to treatment. Sensitivity analyses performed on these coefficients showed no substantive effect on the outcome.

Only the first year following treatment is explicitly modeled in the decision tree. For outcomes after one year, we developed estimates based on observational data and clinical expert consultations for average duration of life based on disease state at the end of one year (labeled “prognosis…at 1 year” in **Table 1**. While the model looked at costs and outcomes specific to the *BRAF*+ patient population, the cost of testing *all* patients was added to the cost estimated for any treatment strategy that included targeted therapy (i.e. vemurafenib).

**Table 1:** 

Input	Base Case	Source
***Clinical Outcomes***		
vemurafenib objective response rate (%)	0.48	9
vemurafenib complete response rate	0.06	8,9
vemurafenib 3 month survival	0.98	8,9
vemurafenib 6 month survival	0.84	8,9
vemurafenib 12 month survival	0.58	8,9
dacarbazine objective response rate	0.05	9
dacarbazine complete response rate	0.00	2,9
dacarbazine 3 month survival	0.78	9
dacarbazine 6 month survival	0.64	9
dacarbazine 12 month survival	0.27	2–4,9
ipilimumab rate of controlafter induction cycle	0.358	6
ipilimumab rate of progressionafter induction cycle	0.642	6
ipi rate of reinduction if progressionafter initial response	0.750	UCSF
ipilimumab rate of progressionafter reinduction	0.250	6
ipilimumab 3 month survival	0.840	6
ipilimumab 6 month survival	0.650	6
ipilimumab 12 month survival	0.44	6
ipilimumab 12 month PFS	0.15	6
Prognosis if progressive diseaseat 1 year (years)	0.250	8,9
Prognosis if stable disease at1 year on vemurafenib (years)	0.333	8,9
Prognosis if partial response at1 year on vemurafenib (years)	0.830	8,9
Prognosis if complete response at1 year on vemurafenib (yrs)	1.500	8,9
Prognosis if stable disease at1 year on ipilimumab (years)	1.000	6,17
Prognosis if partial response at1 year on ipilimumab (years)	5.000	6,17
Prognosis if complete responseat 1 year on ipilimumab (yrs)	9.000	6,17
Prognosis after 3rd line salvage (years)	0.167	UCSF
***Costs***		
Dacarbazine (cycle)	$364.90	UCSF
vemurafenib (month)	$13,020.00	5
Imaging (PET/CT)	$1,200.00	UCSF
Office Visit	$100.00	16
Monitoring Labs	$50.00	16
Neutropenia Hospitalization^12^	$19,110.00	15
SCC removal	$313.18	UCSF
Cobas Test	$150.00	UCSF
Ipilimumab (4-dose induction or reinduction)	$150,577.68	5
3rd-line therapy	$652.96	UCSF
***Health State Utilities***		
Partial response	0.88	19
Stable Disease	0.8	19
Progressive Disease	0.52	19
3rd-line salvage	0.46	UCSF
sympomatic melanoma (penalty)	−0.16	19
Average Side Effects vemurafenib (penalty)	−0.0634	9,19
Average SE dacarbarbazine (penalty)	−0.0364	9,19
Average SE ipilimumab (per cycle)	−0.03	6,19

Source numbers reflect the corresponding citation in the references section of this article. A source of “UCSF” refers to data from the UCSF Medicare reimbursement rates for cost inputs, and consensus among clinicians at the UCSF melanoma center for clinical inputs.

### Inputs

**Table 1** reports all key inputs. We derived clinical outcome inputs for the first year from the phase III randomized-controlled trials instrumental in the approval of vemurafenib and ipilimumab [6], [9]. We used phase II data as well as previous RCTs including dacarbazine to estimate prognosis after the first year of follow-up [2]–[4], [9], [17]. Life expectancy in the absence of disease was based on Centers for Disease Control and Prevention population averages [18]. We based cost inputs for medications on RED BOOK Online [5]. We based all other treatment costs on Medicare reimbursement rates to UCSF and previously-published economic analyses (adjusted to 2013 dollars) [15], [16]. We used health-state utility inputs from a previously-published population-based survey which calculated health utilities for multiple metastatic melanoma disease states and treatment side-effects using the standard gamble method [19]. For side-effects that had no published health utility data (e.g. cutaneous squamous cell carcinoma with vemurafenib), we consulted clinical experts who treat these complications.

We derived QALY values by combining health state utility inputs and clinical probability inputs to determine duration of life and how much time patients would spend in each disease state.

### Analysis

We performed all analyses and built all models in Microsoft Excel Professional Plus 2010 software. We performed one-way sensitivity analyses on all variables in Table 1 within the ranges reported in that table. In most cases, these ranged from 50% to 150% of the base case. We made exceptions for values that are unlikely to vary significantly (e.g. health state utilities representing overall quality of life), values that had the potential to vary more dramatically (e.g. penalties to health-state utilities), and we did not consider medication treatment costs above the current wholesale value. We performed multi-way sensitivity analyses varying several clinical efficacy values simultaneously for both vemurafenib and ipilimumab. We also performed a limited analysis comparing vemurafenib with dacarbazine considering only the first year following treatment initiation because this period has the most substantial clinical data available.

## Results

**Table 2** reports costs and QALYS for dacarbazine, vemurafenib, and vemurafenib followed by ipilimumab in the treatment of *BRAF-*mutated metastatic melanoma. It reports the cost and QALY differences and ICERS comparing dacarbazine with vemurafenib and vemurafenib with vemurafenib followed by ipilimumab. **Table 3** reports costs, QALYS, and cost-effectiveness of vemurafenib compared with dacarbazine in only the first year following treatment. **Table 4** reports the minimum and maximum values tested in sensitivity analyses and the range of results produced by adjusting each variable in one-way sensitivity analyses. The base case input is the value used in the main model which produced our results. The range column reports the highest and lowest values used in sensitivity analyses. The ICER range reports the variation in incremental cost-effectiveness ratios in 2013 US dollars per quality adjusted life-year produced from one-way sensitivity analysis of each variable for the comparison labeled in the column header.

**Table 2:** 

Strategy	QALYs	Increase in QALYs^1^	Cost	Increase in Cost^1^	ICER^1^
dacarbazine only	0.30	N/A	$8,391	N/A	N/A
vemurafenib only	0.72	0.42	$156,831	$148,440	$353,993
vemurafenib + ipilimumab	1.34	0.62	$254,695	$97,864	$158,139

1 These values compare the strategy to the one directly above it on the table.

**Table 3:** 

Strategy	QALYs	Increase in QALYs^1^	Cost	Increase in Cost^1^	ICER^1^
dacarbazine only	0.26	N/A	$7,699	N/A	N/A
vemurafenib only	0.46	0.21	$105,073	$97,375	$471,702

1 These values compare the strategy to the one directly above it on the table.

**Table 4:** 

Input	BaseCase	Range		ICERRange^(vemurafenib)^	ICERRange^(vem+ipi)^
***Clinical Outcomes***		Min	Max		
vemurafenib objective response rate (%)	0.48	0.24	0.72	($310 k–$425 k)	($151 k–$169 k)
vemurafenib complete response rate (%)	0.06	0.03	0.09	($343 k–$367 k)	-
dacarbazine objective response rate (%)	0.05	0.03	0.08	($341 k–$376 k)	-
dacarbazine complete response rate (%)	0.00	0.00	0.01	($353 k–$360 k)	-
ipilimumab rate of controlafter induction cycle (%)	0.358	0.180	0.54	-	($139 k–$162 k)
ipi rate of reinduction ifprogression after initial response (%)	0.750	0.380	1.00	-	($148 k–$179 k)
ipilimumab rate of progressionafter reinduction (%)	0.250	0.130	0.38	-	($149 k–$169 k)
ipilimumab 12 monthprogressionfree survival (%)	0.15	0.08	0.23	-	($148 k–$168 k)
Prognosis if progressivedisease at 1 year (years)	0.250	0.130	0.38	($354 k–$354 k)	($154 k–$161 k)
Prognosis if stable diseaseat 1 yearon vemurafenib (years)	0.333	0.170	0.50	($353 k–$355 k)	-
Prognosis if partial responseat 1 yearon vemurafenib (years)	0.830	0.420	1.25	($343 k–$367 k)	-
Prognosis if complete responseat 1 year on vemurafenib (yrs)	1.500	0.500	5.00	($300 k–$381 k)	-
Prognosis if stable diseaseat 1 yearon ipilimumab (years)	1.000	0.330	2.00	-	($145 k–$168 k)
Prognosis if partial responseat 1 yearon ipilimumab (years)	5.000	1.000	7.50	-	($123 k–$229 k)
Prognosis if complete responseat 1 yearon ipilimumab (yrs)	9.000	1.500	13.50	-	($135 k–$220 k)
Prognosis after3rd line salvage (years)	0.167	0.080	0.25	-	($155 k–$161 k)
Probability of switchingto ipilimumab ifrespond>progress (%)	0.660	0.330	1.00	-	($158 k–$159 k)
% of time receivingvemurafenibafter progression (%)	0.330	0.000	1.00	($327 k–$412 k)	($141 k–$167 k)
***Costs***					
Dacarbazine (cycle)	$364.90	$182	$365	($354 k–$356 k)	-
vemurafenib (month)	$13,020.00	$3,255	$13,000	($90 k–$354 k)	($158 k–$180 k)
Imaging (PET/CT)	$1,200.00	$600	$1,800	($351 k–$357 k)	($157 k–$160 k)
Office Visit	$100.00	$50	$150	($354 k–$354 k)	($158 k–$158 k)
Surveillance Labs	$50.00	$25	$75	($354 k–$354 k)	($158 k–$158 k)
Neutropenia Hospitalization	$19,110.00	$9,000	$29,000	($352 k–$356 k)	-
SCC removal	$313.18	$157	$470	($354 k–$354 k)	($158 k–$158 k)
Ipilimumab(4-dose induction or reinduction)	$150,577.68	$37,000	$150,600	-	($22 k–$158 k)
3rd-line therapy	$652.96	$325	$1,000	-	($158 k–$158 k)
***Health State Utilities***					
Partial response	0.88	0.78	0.98	($328 k–$384 k)	($150 k–$167 k)
Stable Disease	0.8	0.7	0.9	($337 k–$372 k)	($155 k–$162 k)
Progressive Disease	0.52	0.42	0.62	($336 k–$374 k)	($157 k–$159 k)
3rd-line salvage	0.46	0.36	0.56	-	($157 k–$160 k)
sympomatic melanoma(penalty)	−0.16	−0.06	−0.26	($353 k–$355 k)	($154 k–$162 k)
Average SideEffects vemurafenib (penalty)	−0.0634	−0.01	−0.1	($316 k–$386 k)	($157 k–$160 k)
Average SE dacarbarbazine(penalty)	−0.0364	−0.01	−0.1	($323 k–$364 k)	-
Average SE ipilimumab(penalty per cycle)	−0.03	−0.01	−0.1	-	($154 k–$173 k)

PET = positron emission tomography. CT = computed tomography. SCC = squamous cell carcinoma. SE = side effects.

### Impact of prognosis after 1 year

Varying prognosis for the partial response, stable disease, and progressive disease categories did not vary results by more than 10%. Varying prognosis in the complete response group produced ICERs between $381,000 per QALY for a. 5 year additional duration of life ranging to $300,000 per QALY for a five year additional duration of life. For ipilimumab, a sensitivity analysis assuming a patient age of 50 and allowing for an average life expectancy in patients with a complete response reduced the ICER to $90,000 for vemurafenib followed by ipilimumab compared with vemurafenib alone.

### Impact of treatment cost of vemurafenib

Varying treatment cost of vemurafenib produced ICERs between vemurafenib and dacarbazine ranging from $90,000 per QALY for 25% of its current cost up to the base case of $354,000. The threshold of $100,000 per QALY was crossed at a cost of $3600 per month.

### Impact of treatment cost of ipilimumab

Varying the treatment cost of ipilimumab between its current cost and 25% of its current cost produced ICERs ranging between $22,00 and the base case of $158,000. The threshold of $100,000 per QALY gained comparing vemurafenib alone to vemurafenib followed by ipilimumab was crossed at a cost of $102,000 per treatment course of ipilimumab.

### Impact of clinical efficacy in multi-way sensitivity Analyses

Varying multiple key clinical factors for vemurafenib simultaneously (objective response rate, complete response rate, and prognosis after partial and complete response) produced ICERs between $256,000 and $483,000 per QALY for vemurafenib compared with dacarbazine. Varying multiple key clinical factors for ipilimumab simultaneously (induction and reinduction response rates, and prognosis after partial and complete response) produced ICERS between $94,000 and $361,000 per QALY for vemurafenib followed by ipilimumab compared with vemurafenib alone.

### Impact of treatment side effects on quality of life

To assess the maximum potential influence of side effects, we performed an analysis increasing the utility penalty of dacarbazine side effects to 0.2 (20% absolute reduction in quality of life); this produced an ICER of $285,000 when comparing vemurafenib with dacarbazine. Decreasing the utility penalty of vemurafenib side effects to 0 produced an ICER of $309,000. Increasing the per cycle utility penalty of ipilimumab to 0.2 produced an ICER of $199,000 for vemurafenib followed by ipilimumab compared with vemurafenib alone.

## Discussion

*BRAF* testing and targeted treatment in *BRAF-*mutated melanoma patients is not standard of care in all countries and all clinical practices, and understanding the total-cost economics and health value produced from this treatment strategy may be valuable in guiding future standards. This is the most up-to-date economic analysis for dacarbazine in patients with metastatic melanoma, and the only analysis looking at a strategy of sequential therapy of vemurafenib and ipilimumab. The treatment of metastatic melanoma is a fast-changing field, and understanding and quantifying the efficacy and efficiency of treatment options is increasingly important as clinicians are offered more options for their treatment decisions. Further, as high-cost genetic-targeted treatments become standard treatment for more cancers and the proportion of health care resources allocated these treatments grows, the cost-effectiveness of these treatments needs to be transparent and available for decision-makers.

While there is demonstrated uncertainty in the precision of the estimate for the cost-effectiveness of vemurafenib compared to dacarbazine, at its current price, the range of possible ICERs exceeds any threshold suggested as a desirable cutoff for cost-effectiveness [20], [21]. Compared to dacarbazine, vemurafenib demonstrates a clear benefit in both quality and duration of life. However, the current cost of this benefit is higher than the usual cost of treatments to achieve an equal benefit. Dacarbazine is considerably less expensive than vemurafenib, in part because it is off-patent and has a generic form available. Sensitivity analyses revealed that vemurafenib would be cost-effective at a threshold of $100,000 per QALY if the price were to fall to a treatment cost of $3600 per month, and it is certainly plausible that the generic form of vemurafenib will cost less than this value.

Compared to vemurafenib alone, the addition of ipilimumab demonstrates a clear benefit in both quality and duration of life, and proved to be more cost-effective than the comparison between dacarbazine and vemurafenib but still above most desirable thresholds for cost-effectiveness. Despite the high initial treatment cost of ipilimumab, what drives the difference in cost-effectiveness of this strategy is the potential for a durable response which does not require continuous lifetime treatment.

The primary strength of this analysis lies in the quality of its inputs. The majority of clinical probabilities are based on outcomes reported in pivotal, large randomized-controlled trials comparing vemurafenib directly to dacarbazine and ipilimumab to a placebo-equivalent [6], [9]. The health utility inputs come from directly measured utilities specific to metastatic melanoma.

The primary limitation of this analysis is the lack of long-term follow-up in the clinical trials used to approve both vemurafenib and ipilimumab, and consequently a lack of clear data on the prognosis of patients who are still alive after one year of treatment. This limitation was addressed by referencing observational data for long-term outcomes on vemurafenib, dacarbazine, and ipilimumab and by performing sensitivity analyses to quantify the potential effect of this uncertainty.

Additionally, when modeling the outcomes of second-line ipilimumab, this analysis operated on several assumptions which could be false. Most notably, we assumed that the beneficial effects of vemurafenib and ipilimumab when used sequentially are additive, rather than synergistic or redundant. While there is retrospective evidence that the efficacy of ipilimumab does not differ based on BRAF-positivity, and the phase 3 trial used as the primary source for the efficacy of ipilimumab used a patient population who had failed one previous treatment, there is no published evidence about the benefit of ipilimumab following failure of vemurafenib specifically [22].

This model looked only at the direct patient-level clinical benefits of treatment, and does not account for the value of information gained from the development and use of these treatments to inform future treatments for metastatic melanoma, other *BRAF*-mutated cancers, or other mutation-specific targeted therapies.

We did not model the combination of *BRAF* and *MEK* inhibitors due to the current lack of clinical data. Because combination therapy is costly and currently used in clinical practice without definitive evidence for efficacy, we believe a future decision analysis considering the range of potential utility of this treatment strategy would be a valuable way to address this uncertainty.

In conclusion, vemurafenib was found to increase both quality and duration of life when compared with dacarbazine, but at a treatment cost higher than any desirable cost-effectiveness threshold. The addition of ipilimumab to vemurafenib was found to be more cost-effective, but still above most desirable thresholds. The most influential factor in the cost-effectiveness of vemurafenib is treatment cost. Because of the continued poor prognosis of treated patients with metastatic melanoma, there is potential for future treatment strategies to be cost-effective even at a high treatment price if they allow for a durable response without a continuous treatment cost.
