# Correction: Cost-Effectiveness of Treatment Strategies for *BRAF*-Mutated Metastatic Melanoma

**DOI:** 10.1371/journal.pone.0126988

**Published:** 2015-04-20

**Authors:** Patti Curl, Igor Vujic, Laura J. van ‘t Veer, Susana Ortiz-Urda, James G. Kahn

There are errors in the Funding section. The correct funding information is as follows: This study was supported by the National Cancer Institute of the National Institutes of Health under award number K08CA155035 and the Melanoma Research Alliance. The authors are also grateful to Timothy Dattels for his generous support. The funders had no role in study design, data collection and analysis, decision to publish, or preparation of the manuscript.

## References

[1] Curl P, Vujic I, van ‘t Veer LJ, Ortiz-Urda S, Kahn JG (2014) Cost-Effectiveness of Treatment Strategies for *BRAF*-Mutated Metastatic Melanoma. PLoS ONE 9(9): e107255 doi:10.1371/journal.pone.0107255 25198196 10.1371/journal.pone.0107255 PMC4157865

